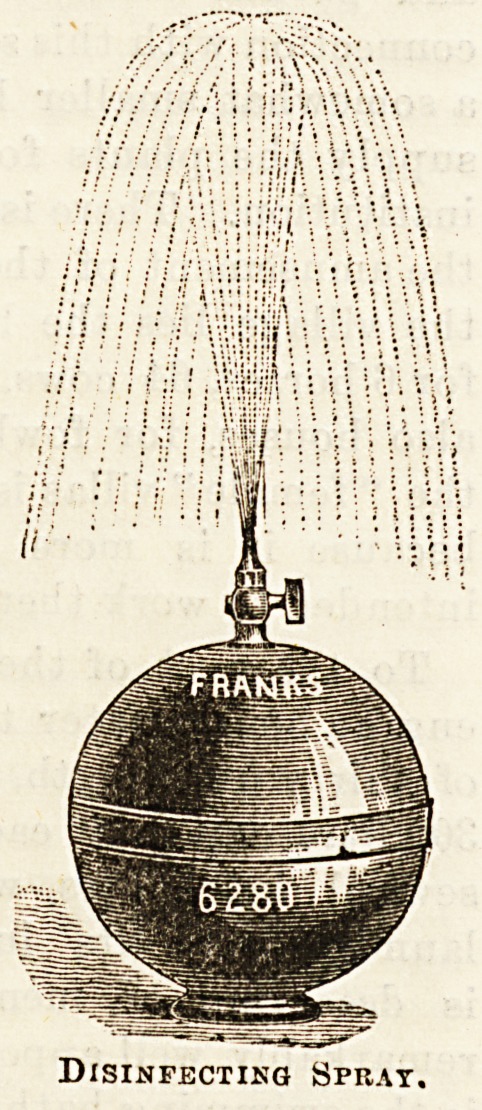# Water Pressure Accumulator

**Published:** 1894-10-20

**Authors:** 


### PRACTICAL DEPARTMENTS.

### WATER PRESSURE ACCUMULATOR.

Messrs. C. and S. Frank, 25, Aldermanbury, E.C., have brought to our notice an ingenious invention of theirs for utilising, as a fountain or spray, the pressure of water in the ordinary main. The apparatus is shown in the accompanying illustration, and will be seen to consist of a globe, which is made of metal plated inside and out, and can be had in various sizes. The principle is very simple. A tube runs from the neck of the globe to almost the bottom, at the top being a tap, above which mouthpieces of different sizes and shapes can be screwed. To fill it a rubber ring is first screwed on and the neck then applied tightly to an ordinary water tap, both vessel and water taps being turned fully on. The water rushing down the tube forces the air into the upper part of the globe, which when thus filled and the taps again turned off will be found to contain water with accumulated pressure equal to the main filled from, and can be carried to any convenient place, where it will discharge it again with the same force. It should be mentioned that after the taps are turned off the rubber ring must be removed, and then any one of the several different mouthpieces can be attached. Our first illustration, which we give by permission of Messrs. Frank, shows a shampoo arrangement. The vessel can be also used as a disinfecting spray as in the second illustration (the disinfectant is introduced by means of a key at the bottom of the globe) or as a fountain or "air cooler" for conservatory or table. The jet will last for a very long time, or longer than would be imagined without a practical trial. As a throat or ear spray it has met with medical approval, and for freshening the atmosphere of the sick room it will be found very effective. Messrs. Frank have also brought out a somewhat similar invention in the form of a syringe, which is intended to be used in the same way, as a disinfecting spray, and possesses very considerable diffusing power.

Shampoo.

Disinfecting Spray.

## Figures and Tables

**Figure f1:**
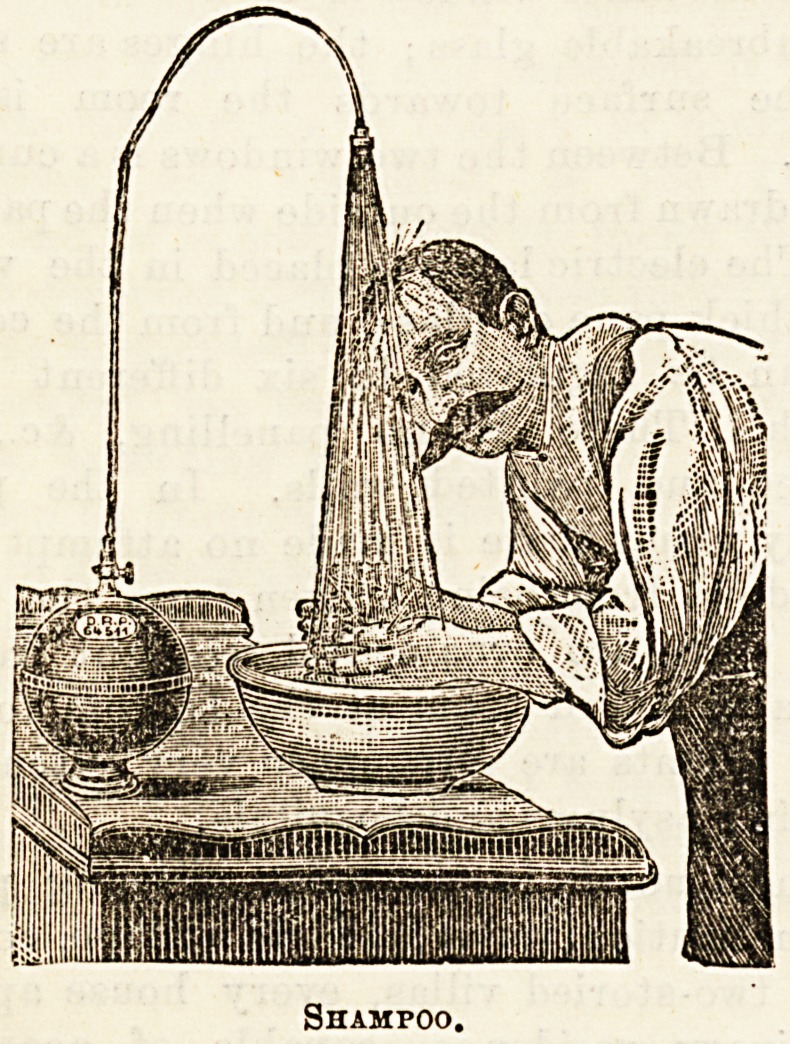


**Figure f2:**